# Beyond Fluid Distribution: Synergistic Bioelectrical Impedance Analysis Metrics for Refined Sarcopenia Screening

**DOI:** 10.31662/jmaj.2026-0230

**Published:** 2026-07-03

**Authors:** Takahiko Nagamine

**Affiliations:** 1 Department of Psychiatric Internal Medicine, Sunlight Brain Research Center, Hofu, Japan; 2 Department of Geriatric Medicine, Shoshido Geriatric Health Services Facility, Hofu, Japan

**Keywords:** bioelectrical impedance analysis, extracellular water to total body water ratio, ECW/TBW, muscle quality, phase angle, sarcopenia screening

## To the Editor

Early detection and intervention in sarcopenia are crucial for managing the health of older adults and require integrated assessment of both muscle quality and quantity to ensure effective clinical outcomes. We read with great interest the study by Hioka et al. ^(1)^ regarding sex differences in the extracellular water to total body water ratio (ECW/TBW). While the authors correctly identify ECW/TBW as a vital indicator of age-related changes, a critical examination of the broader literature suggests that these findings must be contextualized within a more rigorous clinical framework to truly advance sarcopenia diagnostics.

The observation that aging impacts ECW/TBW more significantly in men is not a solitary finding, as recent large-scale evidence has already demonstrated that the appendicular extracellular water to intracellular water ratio increases with age and varies significantly by sex ^(2)^. These sex-specific trajectories are crucial because they may reflect fundamental biological differences in muscle fiber distribution, adiposity, and hormonal influences on fluid regulation. Ignoring these nuances risks misidentifying sarcopenia in women, whose age-related shifts may be more subtle yet equally debilitating, or overdiagnosing men whose fluid shifts might be driven by nonmuscular factors. Furthermore, the clinical threshold for these ratios is already well documented; studies indicate that an ECW/TBW exceeding 0.391 serves as a significant predictor for both sarcopenia and diminished physical performance ^(3)^. To provide a truly impactful advancement, research must move beyond confirming established trends and address the physiological noise inherent in the data.

A major limitation in relying solely on raw water ratios is the failure to account for the confounding effects of systemic disease and pharmacotherapy. In elderly populations, an elevated ECW/TBW often reflects fluid retention from heart failure, chronic inflammation, or the use of specific medications such as diuretics and nonsteroidal anti-inflammatory drugs rather than primary skeletal muscle degradation ^(4)^. Consequently, noninvasive measurement in the future must prioritize the intracellular water compartment, which serves as a far more accurate predictor of cellular health, nutritional status, and actual muscle power in older adults ^(5)^.

In summary, the future of noninvasive sarcopenia assessment lies in a multiparametric approach that integrates segmental bioelectrical impedance, phase angle, and sex-specific hydration metrics while strictly adjusting for clinical comorbidities and the biochemical impact of medications ^(2), (4)^. Only by synthesizing these biological and clinical variables can we ensure that water-derived biomarkers accurately reflect the neurobiological and structural reality of the aging muscle rather than secondary, transient fluctuations in systemic hydration.

## Article Information

### Author Contributions

Takahiko Nagamine contributed to all aspects of this work.

### Conflicts of Interest

None

## References

[1] Hioka A, Akazawa N, Okawa N, et al. Sex differences in age-related changes in the extracellular water-to-total body water ratio among community-dwelling individuals. JMA J. 2026;9(2):495-501. 10.31662/jmaj.2025-0368 PMC13058710 41958617

[2] Iwasaka C, Yamada Y, Nishida Y, et al. Association of appendicular extracellular-to-intracellular water ratio with age, muscle strength, and physical activity in 8,018 community-dwelling middle-aged and older adults. Arch Gerontol Geriatr. 2023;108:104931. 10.1016/j.archger.2023.104931 36701946

[3] Park KS, Lee GY, Seo YM, et al. The relationship between extracellular water-to-body water ratio and sarcopenia according to the newly revised Asian Working Group for Sarcopenia: 2019 Consensus Update. Aging Clin Exp Res. 2021;33(9):2471-7. 10.1007/s40520-020-01766-y 33454925

[4] Lai TF, Park JH, Jang M, et al. Elevated extracellular water to total body water ratio and low phase angle in relation to muscle function in middle-aged and older adults. J Int Soc Sports Nutr. 2025;22(1):2536693. 10.1080/15502783.2025.2536693 PMC12332989 40771175

[5] Hetherington-Rauth M, Baptista F, Sardinha LB. BIA-assessed cellular hydration and muscle performance in youth, adults, and older adults. Clin Nutr. 2020;39(8):2624-30. 10.1016/j.clnu.2019.11.040 31837838

